# Idiopathic Isolated Focal Jaw Tremor Responsive to Botulinum Toxin: A Case Report

**DOI:** 10.7759/cureus.99201

**Published:** 2025-12-14

**Authors:** Rui Peres, Filipe Duarte Godinho, Alexandre Camões-Barbosa

**Affiliations:** 1 Physical Medicine and Rehabilitation Department, Hospital Professor Doutor Fernando Fonseca, Lisbon, PRT; 2 Neurology Department, Unidade Local de Saúde de São José, Lisbon, PRT; 3 Clinical Neurophysiology Unit, Centro Hospitalar Universitário de Lisboa Central, Lisbon, PRT

**Keywords:** bont, botulinum toxin, jaw tremor, orofacial, ultrasound

## Abstract

Jaw tremor is a form of orofacial tremor that can be seen as a symptom of various neurological disorders. Diagnosis and management are hindered when it presents without other neurological symptoms, and there is no consensus regarding the treatment approach. The authors report a case of a 72-year-old female with idiopathic isolated persistent jaw tremor. Diagnostic workup included clinical and electromyographic assessment. A significant therapeutic effect was observed with ultrasound-guided botulinum toxin injections.

## Introduction

A tremor is an involuntary rhythmic oscillatory movement of a body part that is produced by synchronous, alternating contractions of agonist and antagonist muscles, resulting in a nearly symmetrical oscillation around the movement's midpoint [1]. It can occur physiologically in some specific conditions, such as in fear and cold, or as a movement disorder [2]. In fact, tremor syndromes are the most common movement disorder encountered in clinical practice, but only a small percentage seek medical attention.

It may be described using the Movement Disorder Society (MDS) framework, which employs a two-axis classification system. Axis 1 considers (1) historical features, including age at onset, temporal onset and evolution, past medical history, family history, and alcohol or drug sensitivity; (2) tremor characteristics, such as body distribution, activation conditions, and frequency; (3) associated signs, including systemic, neurologic, or subtle 'soft' signs; and (4) additional laboratory tests, such as EMG (electromyography), structural or receptor imaging, and serum or tissue biomarkers. Axis 2 addresses etiology, primarily categorized as acquired, genetic, or idiopathic [3].

According to the MDS classification, a syndrome characterized solely by tremor is defined as an isolated tremor syndrome, with etiology specified separately in Axis 2 [3]. The presence of additional neurological signs would reclassify it as a combined tremor syndrome. When restricted to the jaw, the condition is designated as focal, and in the absence of an identifiable acquired or genetic cause, it is further classified under Axis 2 as idiopathic. Thus, a tremor confined exclusively to the jaw, without an identifiable etiology, is categorized as an idiopathic isolated focal jaw tremor. This is a relatively rare and clinically challenging entity, as it may be an early sign of several conditions and can be difficult to classify and distinguish from other movement disorders [4,5]. In a longitudinal aging study, 5.7% of participants were found to have isolated jaw tremor without other features of tremor syndromes or parkinsonism [4].

Silverdale et al. [5] also proposed a classification of orolingual tremor based on clinical features and etiology. Primary jaw tremor included orthostatic and task- and/or position-specific orolingual tremor, with very few reported cases [5-7]. Secondary jaw tremor included classical essential orolingual tremor, with prevalences ranging from 7.5% to 18% in patients with essential tremor (ET); orolingual tremor associated with dystonia; and parkinsonian orolingual tremor, affecting 17% of patients with Parkinson's disease (PD) [8,9]. The classification also included psychogenic orolingual tremor, drug-induced orolingual tremors, "rabbit syndrome" (predominantly with neuroleptic medication), and other secondary causes like myorhythmia in Whipple's disease [5,6]. In most of these conditions, the jaw tremor is associated with tremors or other abnormal involuntary movements that affect additional body parts [6].

Botulinum neurotoxin (BoNT) has been approved worldwide for use in individuals with movement disorders since its initial approval for the treatment of blepharospasm and hemifacial spasm [10].

The use of BoNT for managing jaw tremor has been published, most often in the context of PD, with generally promising outcomes [11]. However, BoNT therapy for jaw tremor has not yet received approval from either the US Food and Drug Administration or the European Medicines Agency. Published reports on its use in idiopathic isolated focal jaw tremor remain exceedingly rare.

We describe a case of primary idiopathic isolated focal jaw tremor involving multiple masticatory and perioral muscles, which was successfully managed with ultrasound-guided BoNT injections.

## Case presentation

A 72-year-old woman was referred to our neurology clinic with an eight-year history of jaw tremor. Her medical history included essential hypertension and depression, the latter managed with fluoxetine 20 mg daily for the past six years. She reported no prior exposure to antipsychotic medications. Since the onset, the tremor had been persistent, with exacerbations during periods of anxiety. Alcohol or caffeine intake does not have an impact on the tremor. It interfered with eating and drinking and had a considerable negative impact on her quality of life, causing marked social embarrassment.

Clinical assessment

On clinical examination, she presented with a predominantly vertical mandibular tremor, with occasional lateralization movements. The tremor diminished with maximal jaw opening but reappeared immediately upon returning to rest (Video 1).

**Video 1:** Patient's jaw tremor before treatment.

There was a very mild, low-amplitude, bilateral upper limb postural tremor, with no resting or kinetic component. The Archimedes spiral did not reveal features consistent with ET. The tremor remained unchanged despite distraction maneuvers, making a functional etiology unlikely. The rest of the neurological examination was unremarkable, with no signs of tremor in other body segments, dystonia, parkinsonism, or ataxia. Family history was notable for two first-degree relatives who had upper limb and jaw tremors.

A diagnostic workup, including brain computed tomography, complete blood count, thyroid, liver and renal function tests, serum electrolytes, and protein electrophoresis, was unremarkable. A provisory diagnosis of idiopathic isolated focal jaw tremor was established, and the patient started primidone and clonazepam. This treatment yielded no clinical benefit and was poorly tolerated due to gastrointestinal side effects (diarrhea and vomiting).

EMG assessment

She was then referred to our outpatient Neurotoxin Clinic. The needle electromyographic examination showed rhythmic, synchronous activation of the bilateral masseter, temporalis, pterygoideus lateralis, anterior belly of the digastricus, mylohyoideus, and mentalis muscles with a frequency of 8 Hz.

Goals

The patient was considered for BoNT treatment with the primary objective of reducing tremor severity, as assessed by the Patient Global Impression of Change (PGIC) scale. Secondary treatment goals included improvement of liquid intake difficulties and reduction of social embarrassment, each evaluated using a 5-point Likert scale. The Likert scale was defined as follows: 1 = much worse, 2 = worse, 3 = no change, 4 = improved, and 5 = much improved.

Procedure

Ultrasound was used to identify potential muscle targets, after which a Teflon-coated EMG hollow needle connected to an EMG apparatus was inserted to confirm rhythmic burst activity consistent with tremor. Following confirmation, incobotulinumtoxinA (reconstituted in 2 mL of 0.9% sodium chloride; 50 U/mL) was injected under combined ultrasound and EMG guidance into the following muscles: masseter (20 U on the right and 20 U on the left), temporalis (10 U on the right and 10 U on the left), pterygoideus lateralis (10 U on the right and 10 U on the left), anterior belly of the digastricus (10 U on the right and 10 U on the left), mylohyoideus (10 U on the right and 10 U on the left), and mentalis (5 U on the right and 5 U on the left), resulting in a total of 130 U of BoNT.

Outcome assessment and follow-up

At one month post-treatment, the patient reported the onset of improvement approximately two days after injection. She experienced a transient, mild reduction in masticatory strength without functional relevance. Tremor severity showed marked improvement, with a PGIC score of 7 (very much improved). Feeding ability improved substantially, as she was able to drink without spilling (5-point Likert scale = much improved), and subjective social embarrassment was also notably reduced (5-point Likert scale = much improved).

At the six-month follow-up, the patient experienced a recurrence of jaw tremor, though with less severity than before treatment. A second ultrasound-guided injection of 150 U of incobotulinumtoxinA was administered: masseter (20 U on the right and 20 U on the left), temporalis (20 U on the right and 20 U on the left), pterygoideus lateralis (10 U on the right and 10 U on the left), anterior belly of the digastricus (20 U on the right and 20 U on the left), and mentalis (5 U on the right and 5 U on the left). In this adjustment, we considered that the mylohyoideos muscles might not be incapacitating, even though they presented with rhythmic burst activity upon EMG assessment. Also, we increased the doses from 10 U to 20 U in each of the temporalis muscles and in each of the digastricus muscles.

One month after the second injection, the patient was reassessed. Improvement onset occurred approximately two days post-treatment, and she achieved the same scores on all three outcome measures as previously. However, the video assessment showed a reduction in tremor amplitude. No adverse events were reported (Video 2).

**Video 2:** Patient's jaw tremor one month after botulinum toxin treatment.

## Discussion

We described a case of idiopathic isolated jaw tremor successfully treated with BoNT. Our patient presented with an idiopathic isolated focal tremor involving the jaw, according to the MDS classification [3]. As this is a diagnosis of exclusion, other entities were carefully ruled out, namely PD, dystonic tremor, and ET.

Facial (lip and jaw) tremor occurs in approximately one-fifth of PD patients and is associated with older age, female sex, longer disease duration, and more severe motor symptoms [9,12]. However, in the present case, the absence of parkinsonian features, such as bradykinesia, rigidity, or postural instability, makes PD or other neurodegenerative parkinsonian syndromes with jaw tremor unlikely.

Given the eight-year duration of this patient's disease, the probability of subsequently developing parkinsonian features is exceedingly low. This issue was specifically examined by Aslam et al. [4], who analyzed clinical and pathological correlations of jaw tremor in a large longitudinal cohort. Their findings demonstrated that isolated jaw tremor, or jaw tremor associated with non-parkinsonian tremor, rarely progressed to clinically or pathologically confirmed PD (15.4% and 8.8% of cases, respectively, developed parkinsonian disorders). In contrast, participants who exhibited parkinsonism at baseline showed a substantially stronger association with underlying PD pathology.

ET is one of the most common movement disorders, affecting approximately 5% of individuals over 65 years of age [13]. According to the MDS proposed criteria [3], the diagnosis of ET requires a minimum three-year history of bilateral upper limb action tremor, with or without tremor in other regions such as the head, voice, jaw, or lower limbs. This three-year threshold is designed to minimize diagnostic overlap with tremor syndromes that initially resemble ET but later evolve to include additional neurological features, such as dystonia, parkinsonism, or ataxia, which must be absent at the time of diagnosis.

Jaw tremor, reported in 7%-18% of patients with ET, may occur as part of the clinical spectrum [8]. In the present case, jaw tremor is the most prominent manifestation. Although the patient also exhibits a postural upper limb tremor, it is very mild, has no functional impact, and shows no kinetic component. In addition, the Archimedes spiral does not display the characteristic two- to eight-axis pattern typically associated with ET. Taken together, these findings suggest that the upper limb tremor more likely represents an enhanced physiological tremor. Specialized electrophysiological testing could help differentiate a central neurogenic oscillation from the mechanical-reflex oscillation characteristic of physiological tremor; however, such specialized neurophysiological studies are not available at our institution.

Despite normal blood test results, including thyroid function tests, upper limb tremor may have an iatrogenic origin, potentially related to fluoxetine use, as selective serotonin reuptake inhibitors (SSRIs) are recognized causes of medication-induced tremor. A similar association has been described for jaw tremor, with two case reports noting onset within two weeks of paroxetine and citalopram initiation, both SSRIs [14,15]. This possibility could, in principle, be explored by discontinuing the drug. However, in this case, the absence of a temporal association between fluoxetine initiation and the onset of jaw tremor, the sustained stability of the patient's depressive disorder since starting fluoxetine, and the minimal functional impact of the upper limb tremor render drug withdrawal inadvisable for diagnostic purposes.

Tremor in dystonia, or dystonic tremor, could be a possible diagnosis for this patient, as tremor falls within the phenomenological spectrum of isolated dystonia and can be challenging to differentiate from other types of tremor [2]. It is often unilateral, and when bilateral, it tends to be asymmetric [16]. This type of tremor primarily occurs during postural or voluntary movements and may be alleviated by a geste antagoniste or when the patient moves the affected body part in the direction of the dystonia [3,16]. Dystonic tremor frequently develops at or after the onset of dystonia in the affected body part or can involve body parts not affected by tremor [3,17]. In some cases, patients may display focal tremor before dystonia becomes evident [18]. Additionally, patients with focal or segmental tremor may later evolve into dystonia. However, in our case, the eight-year clinical evolution of tremor without accompanying dystonia, the symmetrical bilateral presentation on EMG, and the absence of a geste antagoniste make the diagnosis unlikely, yet not impossible.

Finally, a hereditary cause for this tremor syndrome should be considered, given the family history of two first-degree relatives with a similar phenotype. Nevertheless, a diagnosis of primary idiopathic jaw tremor can still be established irrespective of this etiology (MDS Axis 2). A genetic consultation is advisable to support further diagnostic evaluation.

In parallel with these diagnostic considerations, therapeutic strategies were also explored. The lack of response to primidone and clonazepam, as well as the poorly tolerated side effects, led to the referral of the patient to the Neurotoxin Clinic to evaluate the possibility of BoNT treatment.

BoNT is considered a first-line therapy in primary cranial dystonia (excluding oromandibular) or cervical dystonia [19]. It is also useful for spasticity, hemifacial spasm, temporomandibular joint pain, bruxism, spasticity-associated pain, chronic migraine, trigeminal neuralgia, and other pain conditions associated with nerve compression, neurogenic pruritus, and central poststroke pain [20-32].

Dadgardoust et al. [33] reported success in oromandibular dystonia, with multiple authors reporting effective treatment with BoNT for jaw tremor associated with PD and other conditions, such as hereditary trembling chin [9,34].

We identified two previous reports of BoNT use in isolated jaw tremor, although neither shared the clinical characteristics of our patient. In the report by Tarsy and Ro [35], the patient exhibited a tremor that occurred only during speech (particularly the letter 's') or while attempting to drink from a glass and disappeared during eating or at rest. This was classified as a 'focal position-sensitive tremor' and successfully treated with onabotulinumtoxinA injections into the digastric (10 U bilaterally) and masseter (45 U bilaterally) muscles for a total of 110 U of BoNT.

Gonzalez-Alegre et al. [6] described an isolated high-frequency (14 Hz) jaw tremor that was paroxysmal, occurring two to three times per week and lasting 15 minutes to eight hours, which disappeared with mouth opening or teeth clenching and recurred immediately upon returning to rest. This patient was successfully treated with 30 U of onabotulinumtoxinA injected into each masseter, amounting to a total of 60 U of BoNT.

While this case highlights the therapeutic potential of BoNT for jaw tremor, the optimal method of injection remains an important consideration. Although no study directly compares anatomical surface guidance, EMG guidance, and ultrasound guidance for administering BoNT in jaw tremor, studies focusing on applications in focal spasticity and cervical dystonia demonstrate the superior clinical outcomes of both EMG and ultrasound guidance. These outcomes include pain relief following treatment, improved injection accuracy, and a reduced risk of adverse effects [36,37].

Our patient experienced a significant therapeutic benefit from low-dose BoNT for jaw tremor. The inconvenience of a minor reduction in masticatory strength as an adverse effect may be due to a disproportionately strong response at extrafusal motor endplates despite the low BoNT dose used [38]. Nonetheless, there was no negative impact on feeding and no symptoms of dysphagia, and the patient's global impression of change was maximal.

## Conclusions

We report a case of idiopathic isolated jaw tremor responsive to BoNT. Growing evidence supports the use of BoNT across various movement disorders, where it has been shown to effectively reduce abnormal movements in conditions such as cervical dystonia, spasticity, hemifacial spasm, and blepharospasm. With the usage of EMG and ultrasound guidance, the application of BoNT carries a reduced incidence of adverse effects.

In our patient, BoNT under ultrasound guidance produced a notable therapeutic effect with only minor, transient adverse effects. Further clinical studies are warranted to determine whether these findings are reproducible in patients with similar clinical presentations.
